# Development of Simple Sequence Repeat Markers and Genetic Diversity Evaluation of *Mycocentrospora acerina* in Yunnan Province, China

**DOI:** 10.3390/jof9090944

**Published:** 2023-09-19

**Authors:** Huiling Wang, Kuan Yang, Hongping Huang, Liwei Guo, Xiahong He

**Affiliations:** 1 State Key Laboratory for Conservation and Utilization of Bio-Resources in Yunnan, College of Plant Protection, Yunnan Agricultural University, Kunming 650201, China; huiling930906@outlook.com (H.W.); ykweter@outlook.com (K.Y.); huanghp74@sina.com (H.H.); 2 School of Landscape and Horticulture, Southwest Forestry University, Kunming 650224, China

**Keywords:** genetic diversity, genome, *Mycocentrospora acerina*, *Panax notoginseng*, SSR primers

## Abstract

Round spot is a destructive disease that limits of *Panax notoginseng* production in China. However, the genetic diversity of its etiological agent *Mycocentrospora acerina* has yet to be studied. In this work, firstly, we developed 32 *M. acerina* polymorphic microsatellite markers using MISA and CERVUS 3.0 and selected 14 for further analysis. Then, we studied the genetic diversity of 187 isolates collected from *P. notoginseng* round spot using simple sequence repeat markers and polyacrylamide gel electrophoresis. The genetic diversity ranged from 0.813 to 0.946, with 264 alleles detected at the 14 microsatellite loci. The expected average heterozygosity was 0.897.

## 1. Introduction

Sanqi [*Panax notoginseng* (Burk.) F. H. Chen], a member of the Araliaceae family, is widely distributed throughout the Yunnan Province, China. This species is a valuable traditional Chinese medicinal herb with multiple pharmacological applications [1]. Their ginsenosides and amino acids have a number of positive effects on the circulatory system, cardio-cerebral vascular system, central nervous system, and endocrine system, and reduce inflammation [2,3]. *Panax notoginseng* is a perennial herb with a 400-year cultivation history, and the increasing awareness of its medicinal properties in recent years has increased its cultivation area [4]. However, large-scale cultivation has brought severe diseases during production, such as root rot caused by *Cylindrocarpon destructans* var. Crassum [5]; *Fusarium oxysporum* [6] and *F. solani* [7]; round spot, caused by *Mycocentrospora acerina* [8]; and dark speckle, caused by *Alternaria panax* [9]. During the rainy season, the first symptoms appear on leaves and rapidly spread to the entire plant, causing 20–50% yield losses [10], limiting Sanqi cultivation and production [11].

This disease is characterized by a brown spot with a transparent point in the middle, mainly occurring on the leaves [12]. The causative agent, *M. acerina,* is a quarantine fungus in China [13]. There are 23 species of fungi belonging to the *Mycocentrospora* genus (http://www.speciesfungorm.org/, accessed on 12 April 2022), all of which are plant-pathogenic fungi. Moreover, *M. acerina* infects more than 40 plant species globally, including several economically valuable crops such as *Daucus carota* L. var. sativa Hoffm [14], *Paeonia lactiflora* Pall. [15], and *Acer rubrum* L. [16]. It is worth noting that *M. acerina* can infect nearly 23 kinds of weeds, including *Leptopyrum fumarioides* L., *Galium spurium* L., *Matricaria chamomilla* L., and *Bidens bipinnata* L. [17,18,19].

The genotype is affected by the growth and extinction of the host, gene drift, the environment, and reproductive methods. Therefore, the absolute pathogen population is difficult to define. In actual research, all sampled pathogen populations in a limited time and space are usually analyzed as one population [20]. The genetic structure of a plant pathogen population reflects the evolutionary potential and history of the pathogen [21]. Therefore, the ultimate goal of studying pathogen population genetics is to determine the factors that play a major role in the population evolution of pathogenic fungi and understand the rules of interaction between these evolutionary factors. Throughout the history of agriculture, plant pathogens have had important economic and social impacts on humans. Understanding the genetic diversity of plant pathogens will help people better manage the agricultural ecosystem. The pathogenic fungus population and its genetic structure may change with its living environment [22]. The speed of strain evolution is mainly reflected in the number of genetic variations in the pathogen population. Learning the number of these variants will help judge effective maintenance times of disease prevention measures in agriculture. Pathogen populations with complex genetic structures can often adapt faster to host disease resistance and fungicides. Therefore, understanding the genetic structure variations and distributions of phytopathogenic fungi populations will have important guiding significance for disease resistance breeding, the distribution of disease resistance genes, and the use of fungicides in production. The rapid development of molecular biology and genetics has made the accurate detection of genetic variations in plant pathogen populations a reality [23].

Microsatellites or simple sequence repeat (SSR) are often used to evaluate species’ genetic diversity. The term refers to short tandem repeat sequences characterized by nearly identical lengths and base compositions of the repeat unit, with only minor variations observed in a few genes. This method offers simplicity and time efficiency while demonstrating species specificity, excellent stability, and an enhanced ability to accurately reflect species diversity [24,25,26]. Pathogenic fungal populations exhibit variations across different locations; these variations reflect adaptations brought on by differences in control methods and living environments [27]. Additionally, changes in their genetic structure can lead to loss of resistance in host plants [28]. Therefore, the development of molecular markers for *M. acerina* could offer a more comprehensive genetic basis for *M. acerina* studies, which would enhance efforts to control round spots in Sanqi. Therefore, understanding variations in genetic structure and distributions of plant pathogenic fungi populations could facilitate appropriate disease control strategies [29,30]. In this study, we analyzed the SSR characteristics in the *M. acerina* genome and developed SSR primers from *M. acerina.* Moreover, the effectiveness of the primers in analyzing population genetic diversity structure in *M. acerina* was evaluated.

## 2. Materials and Methods

### 2.1. Strain Isolation and Observation

**Figure 1:**
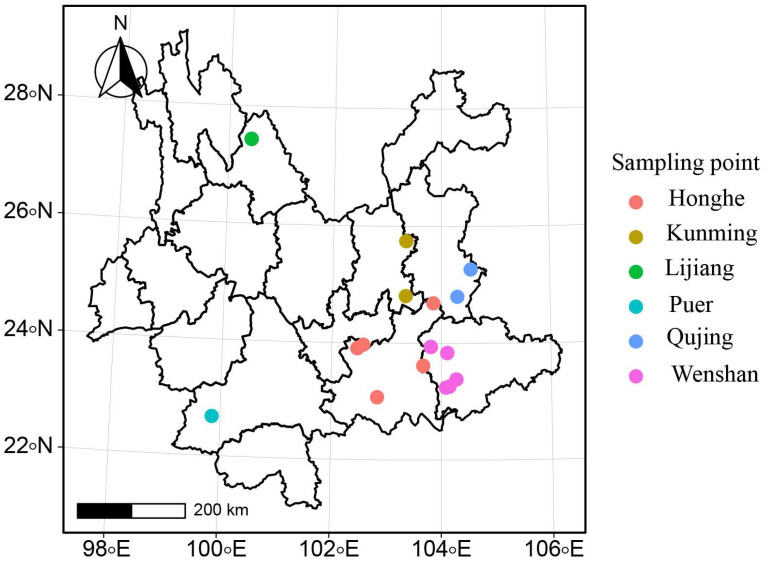
The geographical locations of six *Mycocentrospora acerina* populations in Yunnan.

Leaves from *P. notoginseng* were collected from the six major production regions in Yunnan Province (i.e., Honghe, Wenshan, Qujing, Kunming, Lijiang, and Puer) (Figure 1, Supplementary Table S1). Plant tissue was washed and disinfected before being transferred onto the Potato Dextrose Agar (PDA) medium [31]. Plates were grown at 20 °C for 4 d, and *M. acerina* was identified based on morphological characteristics and internal transcribed spacer (ITS) sequence analysis.

### 2.2. DNA Extraction and Polymerase Chain Reaction Amplification of Internal Transcribed Spacer (ITS1/4) Regions

Genomic DNA was extracted from 0.2 g of mycelium using the Omega Fungi DNA Kit (Kunming Shuoqing Biological Engineering Technology Co. Ltd., Kunming, China) according to the manufacturer’s instructions. Amplification reactions were performed in a 20 μL volume containing 1 μL template DNA, 10 μL mix (DNA polymerase, Buffer, dNTP), 1 μL primer ITS1 (TCCGTAGGTGACCTGCGG), 1 μL primer ITS4 (TCCTCCGCTTATTGATATGC), and 7 μL ddH_2_O. Polymerase chain reaction (PCR) was performed using a T1 thermocycler (Biometra, Göttingen, Germany), with initial denaturation at 94 °C for 5 min, followed by 35 cycles of 94 °C for 45 s, 60 °C for 45 s, and 72 °C for 90 s, and a final extension at 72 °C for 10 min. Amplification products were separated by electrophoresis on 1% agarose gels in a 0.5× TAE buffer, using a 2000-bp DNA ladder as a DNA molecular weight marker. The PCR products were sequenced at Kunming Shuoqing Biological Engineering Technology Co. Ltd. Molecular Evolutionary Genetics Analysis (MEGA 5.1) was used to construct a phylogenetic tree based on the maximum likelihood method. Bootstrap values were evaluated using 1000 replications [32].

### 2.3. Simple Sequence Repeat Screening

Microsatellites were screened using MISA (http://pgrc.ipk-gatersleben.de/misa/, accessed on 18 May 2022) based on the whole genome data of *M. acerina* [33,34]. MISA is a script written in Perl language, which can identify SSRs from genome FASTA files [35]. In the SSR parameter settings, we defined that six, five, four, three, two, and one base were repeated five, five, five, five, six, and ten times.

The genome used in this study was sequenced using Hiseq 2500 from Illumina. After sequencing, two sets of 101 bp long and short paired-end short sequence data were generated. *Mycocentrospora acerina* produced 20.5 Gb of the original sequence after quality control filtering and removing the linker sequences, low-quality sequences, and low-sequence complexity sequences. The K-mer parameter was 75, and the final assembly result was obtained after automatic assembly and gap filling using SOAP de novo software (BGI, Shenzhen, China). The assembled size of *M. acerina* was 39 Mb. The N50 index is an evaluation index of the continuity of genome assembly. This value was calculated by sorting the contig sequence length from the largest to the smallest. The larger the N50 value, the better the continuity of the contig generated by the assembly. In this study, the *M. acerina* genome assembly contig N50 was 151 kb, the scaffold N50 was 567 kb, and the assembly quality was credible.

### 2.4. Primer Design

Microsatellite primers for the whole genome of *M. acerina* were designed in the PRIMER 3.0 (https://primer3.org/, accessed on 28 July 2023) website [36] based on the screened SSR results. Primers were synthesized by Shuoqing Biological Engineering Technology Co. Ltd.

For the initial screening, with 24 isolates from different sources, 118 SSR primers were designed and amplified with 20 μL PCR mixtures. Amplification products were separated on 2.5% agarose gels in 0.5× TAE buffer, using a 50-bp DNA ladder as a DNA molecular marker. Finally, the polymorphism of primers was assessed using CERVUS 3.0 [37], and the primers in which PIC exceeded 0.5 were retained for use in genetic diversity analyses [38].

### 2.5. SSR Analysis of Mycocentrospora acerina Genome

Based on the initial results, 14 primer pairs with high polymorphism in *M. acerina* populations were selected (MP30, MP36, MP47, MP50, MP51, MP56, MP61, MP62, MP63, MP68, MP84, MP92, MP113, and MP114) for use in amplification reactions. Stable and distinct fragments ranging in size from 100 bp to 800 bp were transformed into a binary character matrix (1 = presence, 0 = absence) [39,40].

### 2.6. Statistical Analysis

Genetic diversity parameters of each geographic population, including the observed number of alleles, effective number of alleles, Nei’s diversity index, Shannon’s information index, total gene diversity, intrapopulation gene diversity, the coefficient of gene differentiation, and gene flow [41], were calculated using POPGENE 32 (version 1.32) [42]. The NTSYS-pc (version 2.0) software was used to calculate the genetic similarity coefficient [43]. The phylogenetic tree was analyzed using an unweighted pair-group algorithm with arithmetic averages clustering analysis [44].

## 3. Results

### 3.1. Isolation and Identification of Strains

**Figure 2:**
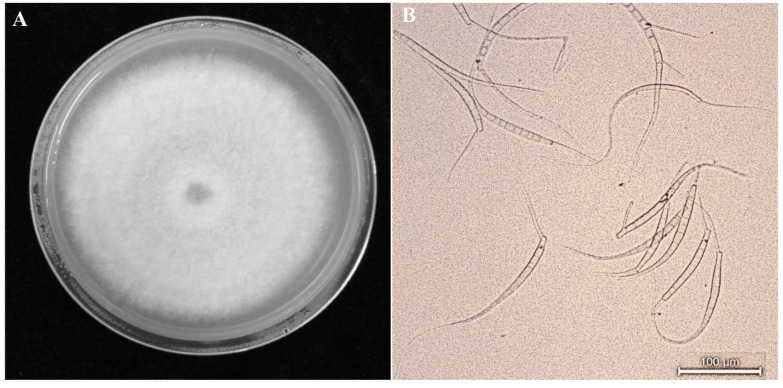
*Mycocentrospora acerina* colonies (**A**), conidia (**B**).

**Figure 3:**
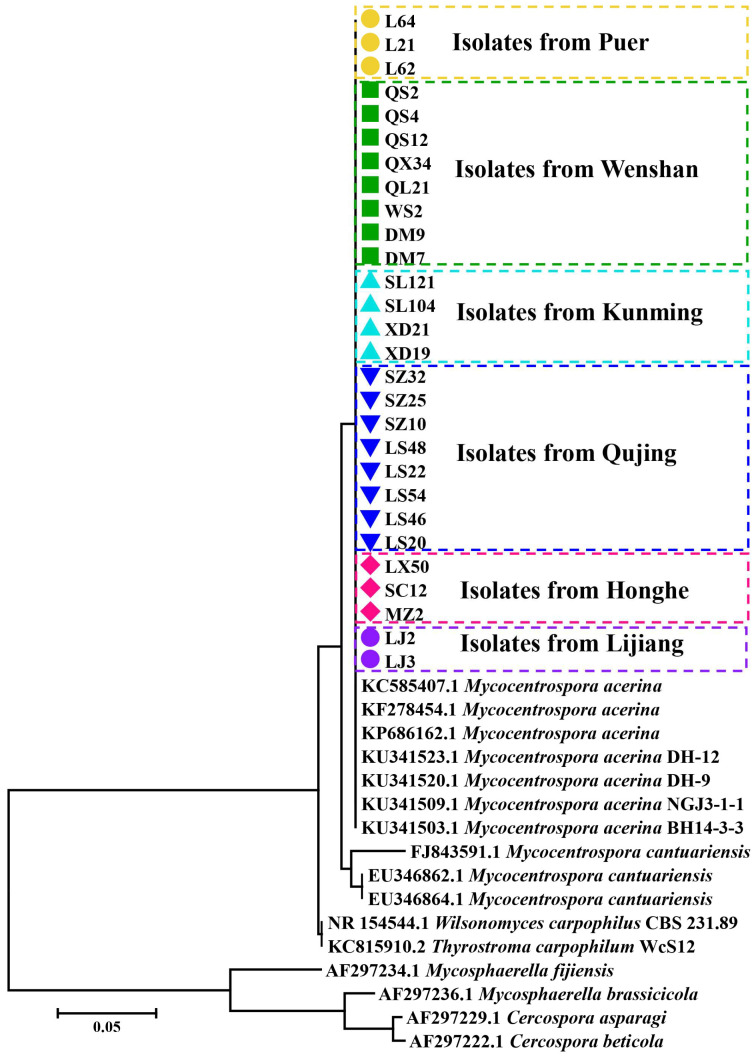
Phylogenetic tree constructed based on internal transcribed spacer 1 (ITS1) sequences.

All isolates were identified based on their colonies/conidium (Figure 2A,B). Phylogenetic trees constructed based on ITS1/4 sequences showed that the isolates were grouped with *M. acerina* (Figure 3).

### 3.2. Genomic SSR Analysis

In the *M. acerina* genome, 8250 microsatellite sequences with 1 to 6 base repeats were obtained. The average length of simple sequence repeats (SSRs) was 26 bp.

**Figure 4:**
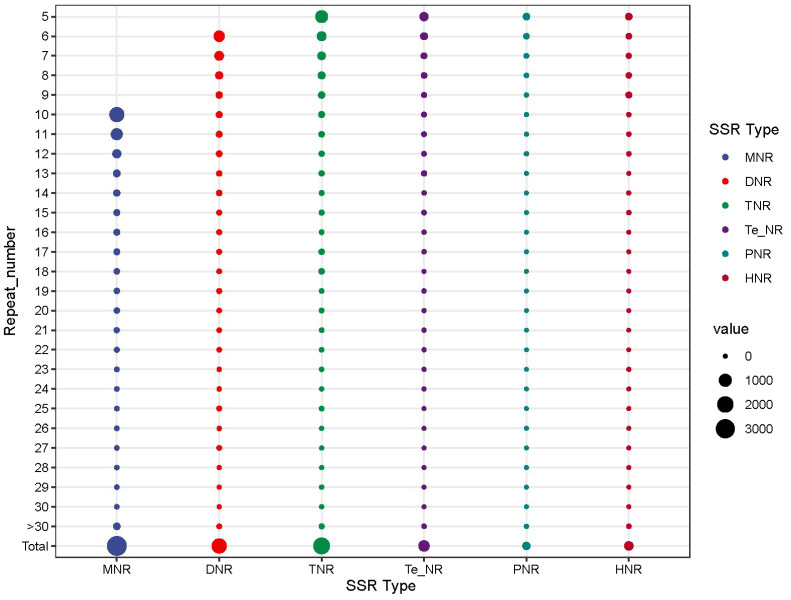
Distributions and frequencies of simple sequence repeats (SSRs) in *Mycocentrospora acerina.* mono-nucleotide repeats, DNR: di-nucleotide repeats; TNR: tri-nucleotide repeats, Te_-_NR: tetra-nucleotide repeats, PNR: penta-nucleotide repeats, HNR: hexa-nucleotide repeats.

Among the SSRs, there were 3379 mono-nucleotide repeats (40.96%), 1608 di-nucleotide (19.49), 2137 tri-nucleotide (25.90%), 641 tetra-nucleotide repeats (7.77%), 179 penta-nucleotide repeats (2.17%), and 306 hexa-nucleotide (3.71%). The maximum repetition times of each SSRs were 81, 40, 155, 217, 72, and 144 (Figure 4).

**Figure 5:**
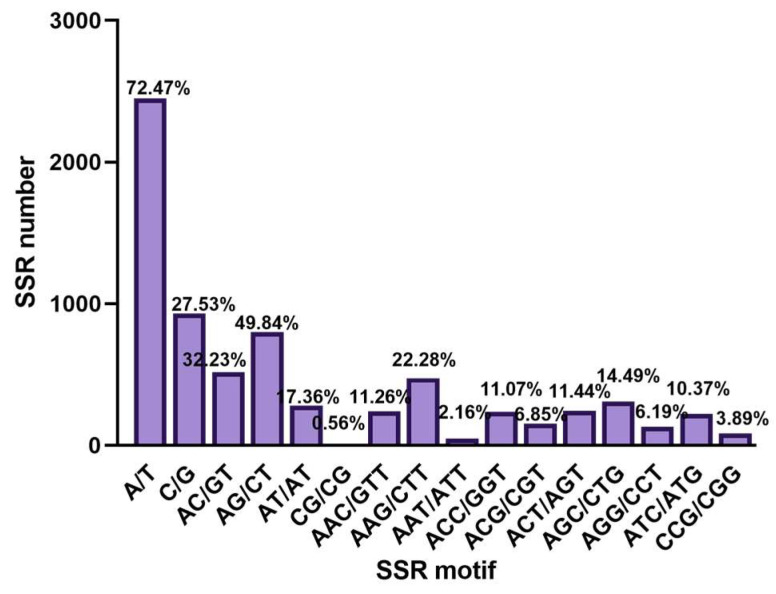
Motifs of mono-, di-, and tri-nucleotide simple sequence repeats (SSRs) in the whole genome of *Mycocentrospora acerina*.

In *M. acerina*, 2449 T or A bases existed in mono-nucleotide SSR (72.47%) and 930 repeats with C or G bases (27.53%). Four types of di-nucleotide repeats were observed: AC (32.23%), AG (49.84%), AT (17.84%), and CG (0.56%). The most frequent types of tri-nucleotide repeats were AAC/GTT (11.26%), AAG/CTT (22.28%), and AAT/ATT (2.16%) (Figure 5).

There were 28 types of tetra-nucleotide repeats, among which ATCC had the highest content (12.48%), and CCCG had the lowest (0.16%). In addition, there were 61 penta-nucleotide repeats, with AACAC (6.7%) and AATCC (5.03%) being the two most common. Hexa-nucleotide repeats were the most abundant (103), with AACCCT being the most common, with 44 motifs (Supplementary Tables S2–S4).

### 3.3. Polymorphism of SSR Primers

Thirty-two pairs of primers had highly polymorphic loci ([PIC] > 0.5) and were used as SSR markers. According to the SSR primer data (Table 1), the average PIC was 0.649, the average allele number per locus was 5.147, the average proportion of locus types was 1.00, and the average expected heterozygosis (He) was 0.721.

### 3.4. Genetic Diversity within Populations

A total of 148 polymorphic bands were amplified from 187 populations of *M. acerina* using 14 SSR primers. The PIC ranged between 0.813 (MP56) and 0.946 (MP114), with an average PIC value of 0.885, all highly polymorphic (Figure S4). The total number is 264 (ranging from 14 on MP61 with the least number and 31 on MP114). The average number of alleles per locus was 18.857. The observed heterozygosis (Ho) of 14 loci was 0, and the expected heterozygosis (He) ranged from 0.831 to 0.951, with an average of 0.897.

### 3.5. Diversity between Populations

Nei’s genetic diversity (0.0896) and Shannon’s information index (0.1712) were higher in the Honghe population (HH), followed by those in the Puer population (LC) (Nei’s genetic diversity = 0.0893 and Shannon‘s information index = 0.1676). They were the lowest in Lijiang (Nei’s genetic diversity = 0.0842 and Shannon‘s information index = 0.143) (Table 2).

The observed allele number (Na) averaged 2.00, while the effective allele number (Ne) averaged 1.11; Nei’s gene diversity (h) was calculated to be 0.0908, and the average value of the Shannon diversity index was found to be 0.1761. The total genetic diversity (Ht) was estimated at 0.0909, with an intra-population genetic diversity (Hs) of 0.0884 and a genetic differentiation index (Gst) of 0.0277, indicating low inter-population genetic variation at only 2.77%. Notably, the estimated level of gene flow (Nm) stood at a substantial value of 17.5757, suggesting that gene flow has significantly contributed to the observed changes in genes across different regions; however, it is not considered the primary factor influencing the population’s genetic diversity. Among the six populations, Kunming and Honghe exhibited the highest genetic similarity (0.9988) and the smallest genetic distance (0.0012). Furthermore, Lijiang and Lancang displayed the lowest genetic similarity (0.9931) and the most significant genetic distance (0.007) (Table 3). Overall, there was minimal genetic divergence between these populations, with a high coefficient of genetic similarity close to 1, indicating a close genetic relationship among strains within each population and low levels of genetic differentiation between populations.

### 3.6. Cluster Analysis

**Figure 6:**
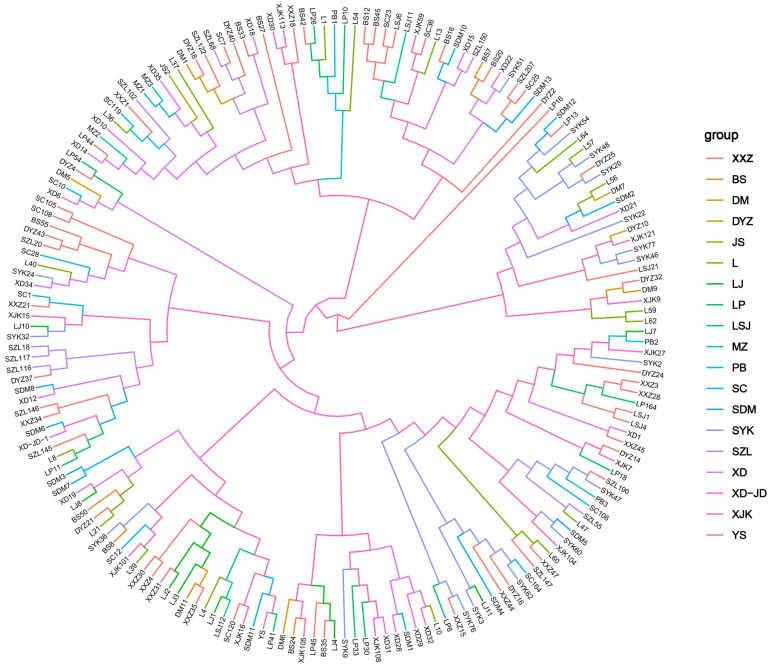
Cluster analysis of 187 *Mycocentrospora acerina* strains in Yunnan province based on the genetic similarity coefficient. Different colors mean different sampling locations. SZL (Jianshui), BS (Luxi), JS (Jianshui), MZ (Mengzi), LP (Longpeng), and SC (Shaochong) were the isolates from Honghe. YS (Yanshan), WS (Wenshan), LSJ (Qiubei), SDM (Qiubei), XXZ (Qiubei), DM (Dumeng), and PB (Wenshan) were the fungus from Wenshan. DYZ and SYK were the isolates from Qujing. XD (Xundian), XD-JD (Xundian), and XJK (Shilin) were the fungus from Kunming. LJ was the fungus from Lijiang.

Cluster analysis revealed that the similarity coefficient ranged between 0.83 and 0.97 (Figure 6).

## 4. Discussion

### 4.1. The Biology of M. acerina and Occurrence Regularity of Round Spot of P. notoginseng

In the 20th century, many studies on *M. acerina* focused mainly on host diversity and transmission methods [13,17]. In contrast, in the 21st century, studies have yet to be conducted on *M. acerina.* Sébastien Louarn studied the influence of *M. acerina* on the polyacetylenes and 6-methoxymellein in organic and conventionally cultivated carrots (*Daucus carota*) during storage [45]. Our laboratory (Key Laboratory of Agricultural Biodiversity and Pest Control of the Ministry of Education) has examined the biological characteristics of *M. acerina*, and much research has been done on the trait of spread in the field. It was found that *M. acerina* is a low-temperature-loving fungus. Its conidia will lyse when the temperature exceeds 32 °C. Its optimum growth temperature is 14–22 °C. The latest measured length of *M. acerina* is (137.36~486.24 μm) × (4.35~16.46 μm) (n = 100), and single conidia can cause infection (Supplementary Figures S1 and S2). *M. acerina* cause initial infection through chlamydospores stored in the soil and spread in the field through conidia on the surface of infected leaves, causing re-infection. Conidia are mainly spread by rain splash. These results show that the genetic distances between the *M. acerina* populations are relatively small, and the similarity is high. This may indicate frequent exchange activities between *M. acerina* from different regions, such as seedlings, cross-regional transportation, and other media dissemination. In addition, *P. notoginseng* round spot prevention and treatment mainly occur in the rainy season (June to September). Using the technology of facility cultivation to build a rain-proof film in the rainy season can prevent *P. notoginseng* round spots and reduce the use of chemical pesticides (Supplementary Figure S3). This reduction in the number of chemical pesticides can then reduce the survival pressure of *M. acerina* and ease the burden of prevention and treatment.

### 4.2. Features of SSR Loci in M. acerina

A total of 8250 repeats were obtained from the screened SSR sequences, indicating that the number of SSRs was higher in the *M. acerina* genome than in some eukaryotes [46,47]. The analysis of microsatellite sequences in *M. acerina* could enhance our understanding of its genome structure, especially the composition of non-coding regions, its mechanisms of pathogenicity, and its heredity at the genome level. Among all SSR types, A and T are abundant, which is consistent with the SSR loci results in most eukaryotic genomes, probably due to the transformation of methylated C residues into T residues [48]. According to Velascor [49], many short repeat sequences indicate a species has a high mutation frequency. In contrast, species with high proportions of long repeat motifs generally have relatively short evolutionary times or low mutation frequencies [49]. Many short repeats of single, dibasic, and tribasic bases were observed in the genome of *M. acerina*, suggesting this *fungus* had a relatively high mutation frequency or a relatively short evolutionary time [50].

With advancements in genome sequencing technologies, molecular marker studies have become more cost-effective [51]. Based on genomic data, we obtained 8250 SSRs, which accounted for 0.55% of the whole genome sequences. In the *Fusarium graminearum* genome, SSR sequences obtained accounted for 0.27% of the whole-genome sequences [52] and 0.21% in the *Sphacelotheca reilianm* genome [53]. In the present study, more than 100 pairs of primers were designed, of which merely 32 pairs were polymorphic, probably because most of the selected primers existed in the coding regions of the genome, with only a few located in the non-coding regions. Studies have demonstrated that SSRs in coding regions often exhibit low polymorphism. These markers should be designed as much as possible within non-coding regions because coding regions have much greater selection pressure than non-coding regions. Moreover, coding regions are relatively conserved during species evolution, while non-coding regions are more likely to evolve or mutate [54,55].

### 4.3. Genetic Diversity of M. acerina in Yunnan

In the current study, the PIC of polymorphic loci ranged from 0.53 to 0.8, which was high compared to the PIC in other eukaryotes. For example, PIC ranged from 0.3 to 0.4 in *Dactylis glomerata* L. [56], from 0 to 0.756 in *Magnaporthe oryzae* [57], and from 0.305 to 0.726 in *Panonychus citri* [58]. According to the results based on primer polymorphism, 14 SSR loci were used to analyze the genetic diversity of *M. acerina* populations. The PIC of screened primers was higher in these loci. However, after population analysis, the genetic diversity of *M. acerina* does not reflect geographic specificity. The potential reason is that the SSR primer loci are within the coding regions of the genome, which have high degrees of conservation [59]. Judicious selection of primers could improve the accuracy of results.

The genetic diversities of *Pyricularia oryzae* Cav. and *Puccina striiformis* f. sp. tritici in Yunnan Province have been reported to be high [60,61]. Therefore, the genetic diversity obtained for *M. acerina* in the study could be due to its background as a quarantine pest in China [62] or its stable survival in areas with highly homogenous ecological environments for prolonged periods. Some of the primary factors influencing the evolution of the population genetic structures of pathogenic fungi include population size, reproductive mode, and genetic drift [20,63,64,65]. This study included 187 *M. acerina* strains with a moderate population. The mode of reproduction of *M. acerina* in the field is asexual reproduction [66], which, to a certain extent, is not conducive to its genetic variation and the evolution of its populations. Pathogenic fungi are small individuals and easily experience genetic drift by natural or artificial means; gene drift is generally considered to hinder the evolution of organisms [67]. In the current study, there were no significant correlations in genetic diversity among strains from different geographical sources. Continuous selection and mutation of pathogenic genes will lead to homozygous individual genes, thus reducing the genetic diversity level of the *M. acerina* population. These factors partly explain that the isolates from the same region cannot be clustered into a group, and some isolates from different regions have very high genetic similarity coefficients. Another possible explanation is that the host *P. notoginseng* is only grown in Yunnan and Guangxi, China, and originates from Wenshan in Yunnan. The rest of the sampling points in the paper have been from *P. notoginseng* planted in the past 5–6 years. Current research shows that, due to the low genetic diversity of sample populations, we can effectively prevent diseases in this area through timely cleaning of diseased leaves, rain-proof cultivation, and alternate use of chemicals [68].

## 5. Conclusions

In this study, we developed 14 SSR primers of *M. acerina* with a good polymorphism that can be used in diversity analysis and identification of *M. acerina* and its closely related species. This result proved that the genetic diversity level of *M. acerina* was relatively low.

## Figures and Tables

**Table 1:** Primers of the whole genome of *Mycocentrospora acerina*.

Locus (Accession Number)	Repeat Motif	Primer Sequence	Size (bp)	Expected Heterozygosity (He)	Polymorphic Information Content (PIC)	Annealing Temperature (Tm)
MP40 (OM824348)	(CTA)_27_	F: CACATGCTCAGTCATTTGTGG	243	0.681	0.599	56
		R: GGTGCAATCGGAAAGAATTG				
MP42 (OM824349)	(TC)_19_	F: AAGCGCACTTGCCTATTGAT	225	0.609	0.53	58.4
		R: GGTGAGTGTTGCTGACGAAA				
MP2 (OM824341)	(CAT)_14_	F: CGTCCATCTTCCTCTTCACC	200	0.71	0.622	56.4
		R: GCTCATGTTCGATGGATGTG				
MP49 (OM824352)	(GA)_25_	F: GGAAGGAAATCCAGGTGTGA	200	0.594	0.505	56
		R: CCCACTTCCTGTTTGCTTGT				
MP54 (OM824356)	(CAA)_19_	F: GTTGTTGCCAGCAAGAGTGA	199	0.623	0.552	58.4
		R: AACAACCCTGGCACTACTCG				
MP55 (OM824357)	(TTGA)_19_	F: TTCCTCTCCCTCTCCCTCTC	214	0.623	0.552	59
		R: ATGCTGCAAGTCTGTTGACG				
MP56 (OM850345)	(TAG)_25_	F: TGTGTGTGTGTTGTTGTTGTTG	228	0.87	0.812	59.5
		R: TGACAAGCAAGTAGATTTTTACGTTT				
MP4 (OM824343)	(GACA)_6_	F: AGGGTAGCTCAAAGCCACTG	273	0.725	0.665	58.4
		R: CTTTCCAAGCTGAGGGTGAG				
MP58 (OM824358)	(GA)_25_	F: TCGTTTTTGGAGCGTTCTTT	209	0.739	0.659	54.4
		R: TGGACGCACTCCTTCTTTTC				
MP52 (OM824355)	(CCA)_11_	F: GCTTCGGTGTCTGGAATCAT	179	0.609	0.53	56.4
		R: AAACTTCAATGTCGCCAAGG				
MP51 (OM824354)	(TTG)_19_	F: CGTCTCTGTTATTGCTGCTTT	159	0.826	0.76	55.7
		R: CGCACAACCAATGAGAAACA				
MP13 (OM824344)	(AGT)_12_	F: CACGTCACGGAGCAAGTAGA	211	0.696	0.622	57.4
		R: TGATGAGGTCCAACGGAGAT				
MP30 (OM824342)	(GT)_13_	F: CATGTGCATTGCTGTGTTGT	170	0.725	0.644	61.4
		R: CAGCGAGTGAATGGAAGTGA				
MP50 (OM824353)	(CTGT)_19_	F: GCTTTACTTTGCCCGTCTGT	200	0.768	0.701	55.4
		R: TGCATCTCCTCACATCCATC				
MP65 (OM824362)	(CA)_22_	F: ACCTCCACACCTGCACCTAC	245	0.609	0.53	59.5
		R: GCGGGCTTGTAGTCGTAGAG				
MP68 (OM824363)	(CAT)_7_	F: GGATATGCCTCACCATTTGC	166	0.797	0.739	55.4
		R: ATATGGAAGGCCGCAGTGTA				
MP83 (OM824364)	(CTT)_12_ (ATGA)_10_	F: TGAGCAGGGGCCAAATACTA	156	0.779	0.702	54.4
		R: TTAAATTCCCATCCCCATCC				
MP36 (OM824346)	(CAT)_17_	F: ATCTGTCACCACCATCACCA	193	0.87	0.812	59
		R: AGCTCGCGATCTAAACATCC				
MP39 (OM824347)	(AGTG)_24_	F: ATGTGTGTGTGTGCCTGGAT	247	0.594	0.505	60
		R: TATATGCCCATTCCCATTCC				
MP46 (OM824350)	(CACT)_10_	F: TTCCTCTGACGCATCCTCTT	207	0.638	0.535	60
		R: TGGGCATGTAATGAGTGGTG				
MP47 (OM824351)	(CAGG)7	F: GATTGTAAGCCGCAGAAGGT	247	0.754	0.68	60
		R: TCACGACTCCATCACTCCAA				
MP20 (OM824345)	(TACA)_11_	F: TGTGTCGCTCACTCACTCAA	239	0.754	0.671	59
		R: GGAAGGAGTGGAGTTGATGG				
MP90 (OM824366)	(TC)_18_	F: TCAAAACCGAAACCCAGAAA	191	0.551	0.503	55.4
		R: GGGAGAAGAAGGGCAGAGG				
MP108 (OM824368)	(TCG)_10_ (TCA)_5_	F: TCACTACCCCTACCCCCTTT	237	0.681	0.599	57.4
		R: CGGTCGGCATAGGGTATTTA				
MP92 (OM824367)	(CTA)_31_	F: ACCCCAACACTCAATCATCC	219	0.71	0.643	54.7
		R: TCTGGCAAGAAGAAGAAATGC				
MP62 (OM824360)	(CTA)_20_	F: CAGAAAATCCTAGCTACTGCTGCT	174	0.725	0.644	56.5
		R: TGCAGTCTCTTCACCCTGTTT				
MP84 (OM824365)	(AGA)_18_	F: TTCAATCGTGCAAGGTGTGT	167	0.739	0.686	58
		R: GAGAGGAGCAGGGCATGTAG				
MP115 (OM824371)	(AC)_7_(TC)_12_	F: TCTGCTGCCATGTAGTGCTC	246	0.768	0.692	55.4
		R: ATGTGATTTTGGGGGAAACA				
MP63 (OM824361)	(TC)_21_	F: CAGACTTCCCAGTCACCACA	195	0.797	0.726	55.5
		R: TTGGCTACTACTGCACCAAAAA				
MP113 (OM824369)	(TG)_7_(AG)_10_	F: CATCTCTCATCTCCCCAGGA	225	0.812	0.746	57.4
		R: AATCCCATCACACGCTTCTC				
MP114 (OM824370)	(CTC)_9_ (TTC)_8_	F: GATGTGCAGAGTTTCGGTCA	232	0.913	0.862	55.4
		R: GGAAGCTGATTCATCCCAGT				
MP61 (OM824359)	(CA)_36_	F: TGGTGGCTAGTTGGTTGGAT	212	0.928	0.878	56.4
		R: GGTCGTCACTGTTGCTTGAA				

**Table 2:** Genetic diversity of the geographic populations of *Mycocentrospora acerina*.

Population	Na	Ne	Nei’s Genetic Diversity	Shannon’s Information Index
Qujing	1.6892	1.1108	0.0888	0.1658
Honghe	1.8041	1.1089	0.0896	0.1712
Kunming	1.7297	1.1103	0.0891	0.1674
Puer	1.6892	1.1099	0.0893	0.1676
Lijiang	1.4122	1.1187	0.0842	0.143
Wenshan	1.777	1.1099	0.0893	0.1693

**Table 3:** Nei’s genetic identity (upper diagonal) and genetic distance (lower diagonal) of the geographic populations of *Mycocentrospora acerina*.

popID	Honghe	Lijiang	Puer	Kunming	Wenshan	Qujing
Honghe		0.9938	0.9976	0.9988	0.9982	0.9977
Lijiang	0.0062		0.9931	0.9937	0.9959	0.9946
Puer	0.0024	0.007		0.9975	0.998	0.9978
Kunming	0.0012	0.0064	0.0025		0.9979	0.9975
Wenshan	0.0018	0.0041	0.002	0.0021		0.9983
Qujing	0.0024	0.0054	0.0022	0.0025	0.0017	

## Data Availability

Not applicable.

## Supplementary Materials

The following supporting information can be downloaded at https://www.mdpi.com/article/10.3390/jof9090944/s1, Supplementary Figure S1: (A) Conidia of *M. acerina* artificially induced in a petri dish, the size of the conidia is (137.36~486.24 μm) × (4.35~16.46 μm) (n = 100). (B) The diseased spots of *P. notoginseng* leave four days after inoculation with single conidia. (C) Early lesions of round spot disease of *P. notoginseng*. Supplementary Figure S2: (A) The diseased spots 72 h after the inoculation of *P. notoginseng* leaves were sprayed with 5 × 10^3^ CFU/mL conidia suspension. (B) The lesions are magnified with an optical microscope. (C) The inoculated *P. notoginseng* leaves were stained with a fluorescent dye (Calcofluor white stain). Conidia can be seen on the lesion under the fluorescence microscope (Leica DM 2000). (D) Conidia produced at the site of a diseased spot, bar = 100 μm. Supplementary Figure S3: (A) *P. notoginseng* with a serious incidence of round spot disease without shelter from rain. (B) *P. notoginseng* cultivated in shelter from rain is growing well, and there is almost no occurrence of disease. We first developed *M. acerina* polymorphic microsatellite markers using *CERVUS 3.0.* Supplementary Figure S4: Representative result amplification pattern generated in part of *Mycocentrospora acerina* samples. Lanes M = DL50 marker; lanes 1 to 32 = partial *M. acerina* isolates. Supplementary Table S1: *Mycocentrospora acerina* populations examined in the simple sequence repeat analysis. Supplementary Table S2: Tetra-nucleotide simple sequence repeat (SSR) motifs in the whole genome of *Mycocentrospora acerina*. Supplementary Table S3: Penta-nucleotide simple sequence repeat (SSR) motifs in the whole genome of *Mycocentrospora acerina*.
