# Supplementary material for: Development of Simple Sequence Repeat Markers and Genetic Diversity Evaluation of Mycocentrospora acerina in Yunnan Province, China
Source: J Fungi (Basel). 2023 Sep 19;9(9):944. doi: 10.3390/jof9090944 (PMC10532959; doi:10.3390/jof9090944)
Supplement: Supplementary file 1 [file jof-09-00944-s001.zip › jof-2567527-supplementary.pdf]

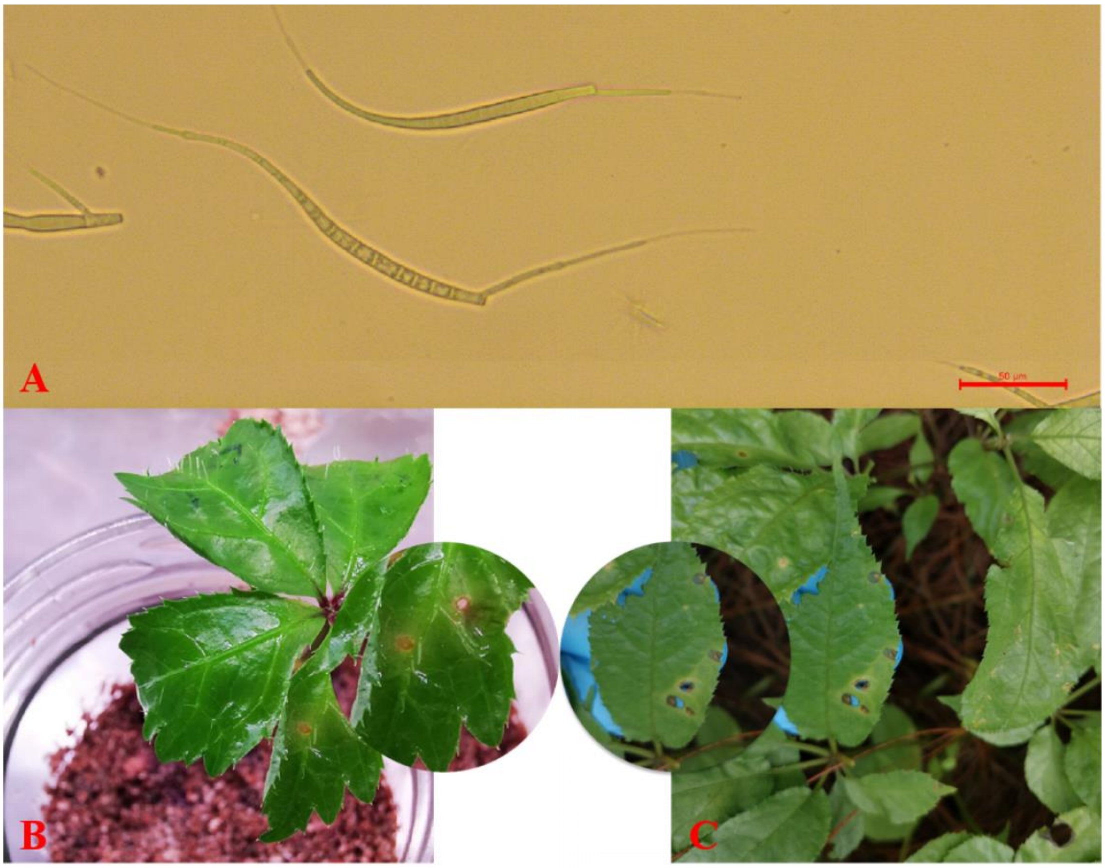

Supplementary Figure 1 A: Conidia of *M.acerina* artificially induced in a petridish, the size of the conidia is (137.36~486.24μm) × (4.35~16.46μm) (n=100). B: The diseased spots of *P. notoginseng* leaves 4 days after inoculation with single conidia. C: Early lesions of round spot disease of *P. notoginseng*.

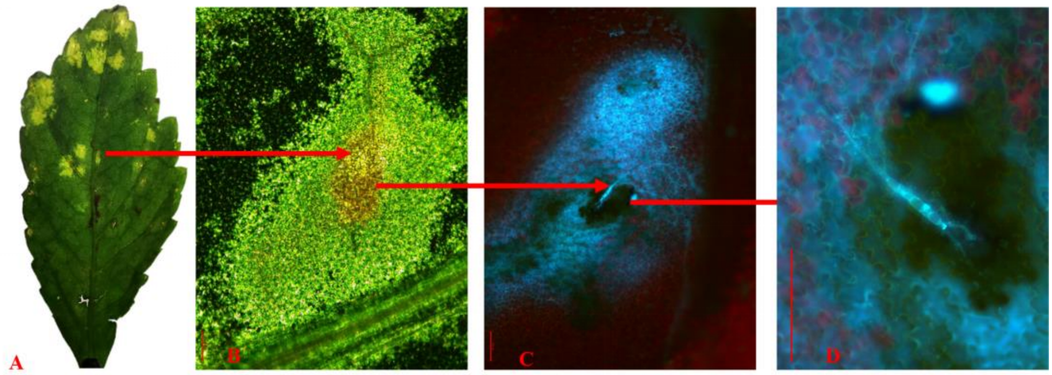

Supplementary Figure 2 A: The diseased spots 72h after the inoculation of *P. notoginseng* leaves were sprayed with $5*10^3$CFU/mL conidia suspension. B: the lesions magnified with an optical microscope. C: The inoculated *P. notoginseng* leaves were stained with fluorescent dye (Calcoflour white stain). A conidia can be seen on the lesion under the fluorescence microscope (Leica DM 2000). D: Conidia produced at the site of a diseased spot, bar=100μm.

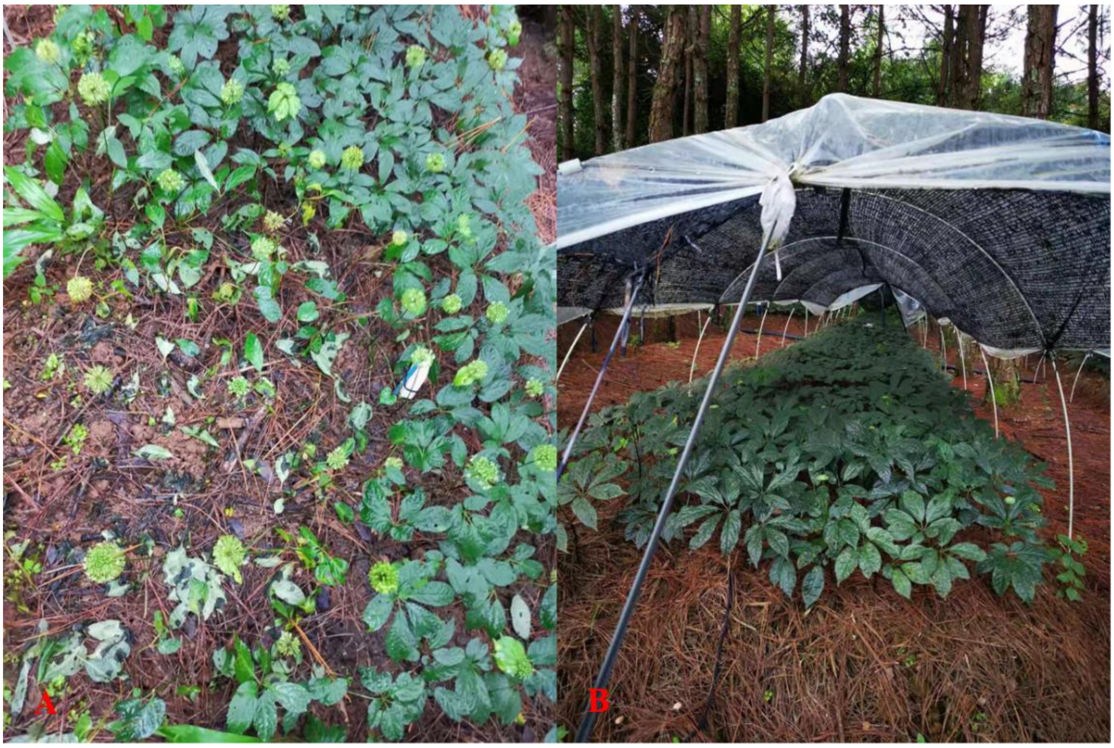

Supplementary Figure 3 A: *Panax notoginseng* with serious incidence of round spot disease without shelter from rain. B: *P. notoginseng* cultivated in shelter from rain is growing well, and there is almost no occurrence of disease.

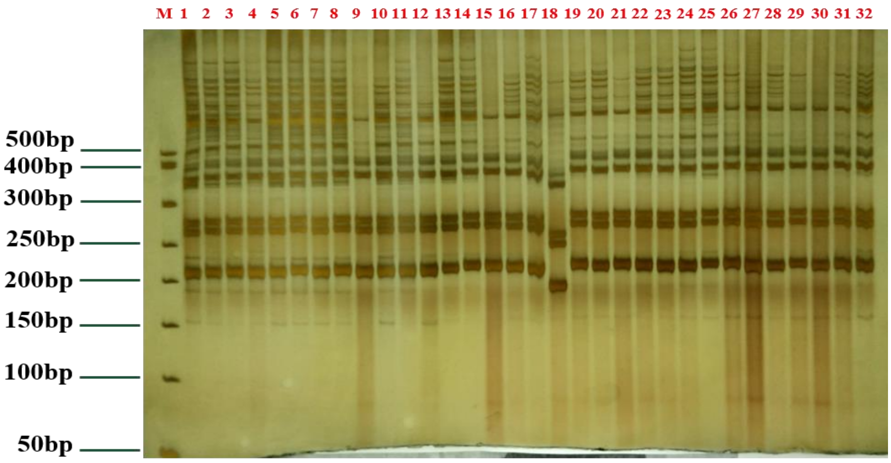

Supplementary Figure 4 Representative result amplification pattern generated in part of *Mycocentrospora acerina* samples. Lanes M = DL50 marker; lanes 1 to 32 = partial *M. acerina* isolates.

Supplementary table 1 *Mycocentrospora acerina* populations examined in the simple sequence repeat analysis.

Location	ID	Population	Isolates	Latitude (N)	Longitude (E)
Honghe (HH)	SC	Shaochong, Honghe	14	23° 54′ 30″	102° 27′ 53″
	LP	Longpeng, Honghe	14	23° 58′ 12″	102° 34′ 13″
	JS (SZL)	Jianshui, Honghe	15	23° 24′ 22″	102° 49′ 58″
	LX (BS)	Luxi, Honghe	13	24° 41′ 14″	103° 51′ 10″
	MZ	Mengzi, Honghe	3	23° 37′	103° 40 ′
Puer (PE)	L	Lancang, Puer	18	22° 40′ 29″	99° 50′ 49″
Wenshan (WS)	YS	Yanshan, Wenshan	1	23° 23′ 4″	104° 16′ 24″
	WS (PB)	Wenshan, Wenshan	3	23° 14′ 30″	104° 5′ 0″
	DM	Dumeng, Wenshan	6	23° 15′ 42″	104° 8′ 42″
	QL (LSJ)	Qiubei, Wenshan	6	23° 56′ 25″	103° 48′ 21″
	QX (XXZ)	Qiubei, Wenshan	14	23° 50′ 2″	104° 6′ 10″
	QS (SDM)	Qiubei, Wenshan	12	23° 50′ 23″	104° 6′ 34″
Qujing (QJ)	SZ (DYZ)	Shizong, Qujing	13	24° 75′ 44″	103° 91′ 78″
	LS (SYK)	Luoping, Qujing	17	24° 47′ 47″	104° 17′ 36″
Kunming (KM)	SL (XJK)	Shilin, Kunming	12	24° 48′ 31″	103° 38′ 16″
	XD	Xundian, Kunming	18	25° 44′ 48″	103 ° 21′
Lijiang (LJ)	LJ	Lijiang	8	26°86'	100°25'

Supplementary table 2 Tetra-nucleotide simple sequence repeat (SSR) motifs in the whole genome of *Mycocentrospora acerina*.

**Motif**	**Number**	**Percentage (%)**	**Motif**	**Number**	**Percentage (%)**
AAAC	16	2.5	ACCT	45	7.02
AAAG	32	4.99	ACGC	4	0.62
AACC	24	3.74	ACGG	1	0.16
AACT	7	1.09	ACTG	29	4.52
AAGG	44	6.86	AGCC	13	2.03
AAGT	6	0.94	AGCG	3	0.47
AATC	67	10.45	AGCT	2	0.31
AATG	19	2.96	AGGC	14	2.18
ACAG	35	5.46	AGGG	14	2.18
ACAT	52	8.11	ATCC	80	12.48
ACCC	2	0.31	ATGC	7	1.09
ACCG	6	0.94	CCCG	1	0.16

Supplementary table 3 Penta-nucleotide simple sequence repeat (SSR) motifs in the whole genome of *Mycocentrospora acerina*.

**Motif**	**Number**	**Percentage (%)**	**Motif**	**Number**	**Percentage (%)**	**Motif**	**Number**	**Percentage (%)**
AAAAC	3	1.68	AAAGG	2	1.12	AACAG	1	0.56
AAAAG	2	1.12	AAATC	4	2.23	AACCT	2	1.12
AAAAT	1	0.56	AAATG	1	0.56	AACGC	2	1.12
AAACT	1	0.56	AACAC	12	6.7	AACTC	2	1.12
AACTG	2	1.12	ACATC	5	2.79	AGAGC	2	1.12
AAGAC	5	2.79	ACATG	1	0.56	AGAGG	4	2.23
AAGAG	4	2.23	ACCAT	3	1.68	AGATC	2	1.12
AAGGC	3	1.68	ACCCT	3	1.68	AGATG	9	5.03
AAGGG	3	1.68	ACCGC	1	0.56	AGCAT	1	0.56
AAGGT	1	0.56	ACCTC	3	1.68	AGCCC	1	0.56
AAGTC	2	1.12	ACCTG	1	0.56	AGGAT	2	1.12
AAGTG	2	1.12	ACGAG	2	1.12	AGGGC	6	3.35
AATCC	9	5.03	ACGAT	1	0.56	AGGGG	3	1.68
AATCG	1	0.56	ACGCC	2	1.12	ATCCC	5	2.79
AATGG	4	2.23	ACGTC	2	1.12	ATCGC	2	1.12
AATGT	2	1.12	ACTAT	2	1.12	ATGCC	3	1.68
AATTC	5	2.79	ACTCC	1	0.56	ACAGG	4	2.23
ACACC	5	2.79	ACTCG	2	1.12	ACAGC	3	1.68
ACACG	1	0.56	ACTCT	2	1.12	ACTGG	6	3.35
ACACT	4	2.23	ACTGC	1	0.56			

Supplementary table 4 Hexa-nucleotide simple sequence repeat (SSR) motifs in the whole genome of *Mycocentrospora acerina*

Motif	Number	Percentage (%)	Motif	Number	Percentage (%)	Motif	Number	Percentage (%)
AAAAAC	1	0.33	AACATG	1	0.33	AAGGGC	2	0.66
AAAACC	1	0.33	AACCAC	3	0.99	AAGGGG	1	0.33
AAAACG	1	0.33	AACCAG	1	0.33	AAGGTG	2	0.66
AAAAGG	1	0.33	AACCCT	44	14.47	AAGTAC	1	0.33
AAAATC	1	0.33	AACCGC	1	0.33	AAGTGG	7	2.30
AAAATG	1	0.33	AACGAC	1	0.33	AATACC	1	0.33
AAACGG	1	0.33	AACGAG	1	0.33	AATACT	2	0.66
AAAGCC	1	0.33	AACGGC	1	0.33	AATAGC	5	1.64
AAAGGG	1	0.33	AACTAC	4	1.32	AATCAC	3	0.99
AAATAG	1	0.33	AACTAG	1	0.33	AATCAG	1	0.33
AAATCC	2	0.66	AACTAT	1	0.33	AATCCC	1	0.33
AAATGC	1	0.33	AACTTC	1	0.33	AATGAC	3	0.99
AAATGG	2	0.66	AAGAGG	11	3.62	AATGAG	3	0.99
AAATGT	2	0.66	AAGAGT	2	0.66	AATGGC	2	0.66
AACAAG	1	0.33	AAGATG	4	1.32	AATGTG	5	1.64
AACACC	2	0.66	AAGCAC	1	0.33	ACACAG	1	0.33
AACACT	1	0.33	AAGCAG	2	0.66	ACGATG	1	0.33
AACAGC	5	1.64	AAGGAC	2	0.66	ACGCCC	2	0.66
AACATC	5	1.64	AAGGAG	3	0.99	ACGTCC	1	0.33
ACACAT	16	5.26	ACTAGC	13	4.28	ACCTCC	2	0.66
ACACCC	1	0.33	ACTCAT	1	0.33	ACCTCT	1	0.33
ACACCT	2	0.66	ACTCCC	1	0.33	ACCTGC	8	2.63
ACACGC	1	0.33	ACTCCG	1	0.33	ACGACT	1	0.33
ACACTC	1	0.33	ACTCCT	2	0.66	AGGGCG	1	0.33

Motif	Number	Percentage (%)	Motif	Number	Percentage (%)	Motif	Number	Percentage (%)
ACACTG	1	0.33	ACTCTC	4	1.32	ATATCC	1	0.33
ACAGGC	1	0.33	ACTCTG	1	0.33	ATCCCC	1	0.33
ACAGTG	1	0.33	ACTGAG	1	0.33	ATCGCC	13	4.28
ACATCC	1	0.33	ACTGCC	4	1.32	ACCCTC	2	0.66
ACATGC	2	0.66	ACTGCG	2	0.66	ACCGTC	2	0.66
ACATGG	1	0.33	ACTGCT	3	0.99	AGCCTG	1	0.33
ACCACT	3	0.99	ACTGGG	1	0.33	AGGATG	12	3.95
ACCAGC	17	5.59	AGAGAT	2	0.66	ACCCAG	1	0.33
ACCATC	2	0.66	AGAGCC	2	0.66	ACCCGC	3	0.99
ACCATG	1	0.33	AGAGGC	2	0.66			
AGAGGG	5	1.64	AGATGC	1	0.33			
